# Scientific literature landscape analysis of researches on oxidative stress in intervertebral disc degeneration in web of science

**DOI:** 10.3389/fmolb.2022.989627

**Published:** 2022-08-11

**Authors:** Yunzhong Cheng, Honghao Yang, Yong Hai, Lijin Zhou

**Affiliations:** Department of Orthopedic Surgery, Beijing Chao-Yang Hospital, Capital Medical University, Beijing, China

**Keywords:** oxidative stress, intervertebral disc degeneration (IDD), bibliometric analysis, VOSviewer, citespace

## Abstract

Oxidative stress plays a significant role in the development of disc degeneration and has attracted widespread attention since it was first researched in 2007. Our study aims to analyze the scientific output of oxidative stress in intervertebral disc degeneration (IDD) and drive future research into new publications. Publications focused on this topic were retrieved from the SCI-EXPANDED (SCI-E) of the Web of Science (WOS) core collection database and were screened according to the inclusion criteria. Bibliometric website, VOSviewer, and Citespace software were used to evaluate and visualize the results, including annual publications, citations, authors, organizations, countries, research directions, funds, and journals. As of 16 February 2022, a total of 289 original articles and reviews were included, and the overall trend of the number of publications rapidly increased. China and the United States were the leading countries for research production in worldwide. The retrieved 289 publications received 5,979 citations, with an average of 20.67 citations and an H-index of 40. The most high-yield author, organization, country, research direction, fund, and journal were Wang K from Tongji Medical College, Huazhong University of Science Technology, China, Cell Biology, National Natural Science Foundation of China, Oxidative Medicine and Cellular Longevity, respectively. The majority of most common keywords were related to the mechanisms and regulatory networks of oxidative stress. Furthermore, with accumulating evidence that demonstrates the role of oxidative stress in IDD, “mitochondria” and “senescence” are becoming the new research focus that should be paid more attention to.

## Introduction

With the aging of society, shoulder and neck pain or low back pain has become a common disease for middle-aged and elderly people, causing a significant economic burden to the whole society (Pulickal et al., 2019). Intervertebral disc degeneration (IDD) is one of the leading causes of shoulder and neck pain or low back pain, and its etiology is complex and multi-factorial, including aging, mechanical stress, smoking, infection, trauma, and genetics (Risbud And Shapiro, 2014). The degenerated intervertebral discs could exhibit the appearance of annulus fibrosus fissures, the loss of nucleus pulposus collagen and water, and the structural changes such as calcification of the vertebral endplates (Cao et al., 2022). However, the mechanism of intervertebral disc degeneration is still unclear and a hotspot for researchers.

Bibliometric analysis and visualization are more effective methods to assess the thematic development of structural contents and could help researchers comprehensively understand the Frontier trends (Dong et al., 2022; Lawson McLean, 2019; Zheng and Wang, 2019). Science Citation Index Expanded (SCI-E) of Web of Science (WOS) Core Collection Database is nowadays widely recognized as an important tool for scientific statistics and scientific evaluation (Xu et al., 2017). Thanks to the quantitative construction of this database and the qualitative contribution of the bibliometrics, most cited publications, top high-yield countries, organizations, authors, research directions, funds, and journals, can be comprehensively evaluated.

However, no bibliometric study on oxidative stress in IDD has been reported. Our study aims to outline the intellectual connections within the dynamic changing of scientific knowledge in the field of oxidative stress in IDD by making good use of the citation database (SCI-E) and the software tools (https://bibliometric.com/, VOSviewer, and Citespace). These results could benefit scholars by better understanding future research directions and offering enough evidences for policy-making.

## Materials and method

### Data retrieving

The Literature data were retrieved through the SCI-E of WOS Core Collection Database in Capital Medical University Library. The search query was “[TS=(Oxidative Stress OR Antioxidative Stress) AND TS=(Intervertebral Disc Degeneration OR IVD Degeneration OR Disc Degeneration)] AND LA=(English) AND DT=(Article or review).” Timespan = From 1 January 2007 to 31 December 2021 (since publications on oxidative stress in IDD started from 2007). The literature searching was accomplished within the same day to avoid bias due to database updates on 16 February 2022. The records were exported by “full records and cited references” in plain text file format and tab-delimited file format, respectively.

### Bibliometric analysis

The trends of publications and citations were charted annually. The contribution of all countries by publications was demonstrated using a pie chart. The total number of publications, the sum of total citations, and H-index of the top ten countries were also obtained. The top 20 most cited articles were recorded and analyzed, including first author, article title, journal of publication, year of publication, the total number of citations, and average citations. The top five records, h-index, total citations, and average citations for authors, organizations, and countries were tabulated directly. Meanwhile, the top ten research directions, funds, and journals with the most publications were charted.

The distribution of the bibliographic records per year of top ten countries, the co-authorship analysis of countries or regions, the distribution of the bibliographic keywords per year of the top ten countries, were analyzed by the bibliometric website (https://bibliometric.com/). The co-authorship relations in the analysis units of authors and organizations, the co-citation analysis of references, journals, and author, the keywords co-occurrence visualization, were all mapped by VOSviewer-1.6.11 software (Nees Jan van Eck and Ludo Waltman, 2019).

Co-citation timeline of references by keywords, keywords clusters on oxidative stress in IDD, the top 25 references with the strongest citation bursts and the top 6 keywords with the strongest citation bursts, as well as details of ten clusters, were Visualized by CiteSpace_5.8. R3 edition (Chaomei Chen, 2003–2022). The time-slicing was selected from January 2007 to December 2021. Years per slice was picked by one. The rest of the parameters were chosen by default setting. The reference was selected for co-citation timeline and burst analyses. The keyword was selected for burst analyses, and details of clusters with three different algorithms were exported into a table.

## Results

### Publication outlines

**FIGURE 1:**
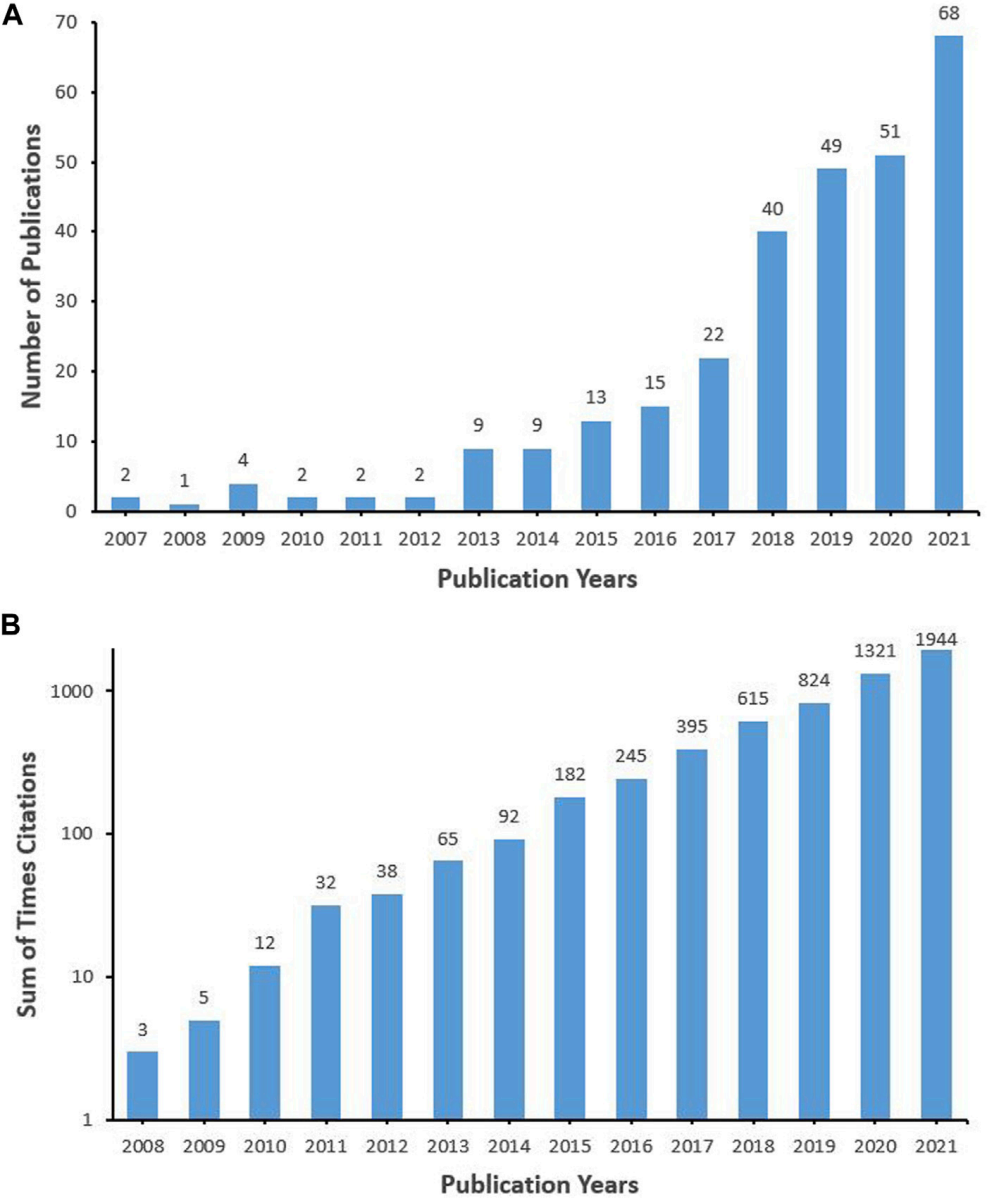
Annual publications **(A)** and sum of times cited per year **(B)** on oxidative stress in intervertebral disc degeneration. (Numbers above the histogram represent the publications and citations, respectively.)

A total of 289 articles and reviews were retrieved in the SCI-E of WOS Core Collection Database from 1 January 2007, to 31 December 2021, with a sum of 5,979 times cited, average citations of 20.67 per item, and an h-index of 40. Figure 1 showed the annual publications and sum of times cited per year on oxidative stress in IDD. The number of publications showed a fluctuating increase year by year and peaked in 2021 (*n* = 68). In addition, the citations started in 2008 (*n* = 3) and increased linearly year by year. The year with the most times cited was 2021 (*n* = 1944).

### Publication by different countries

**FIGURE 2:**
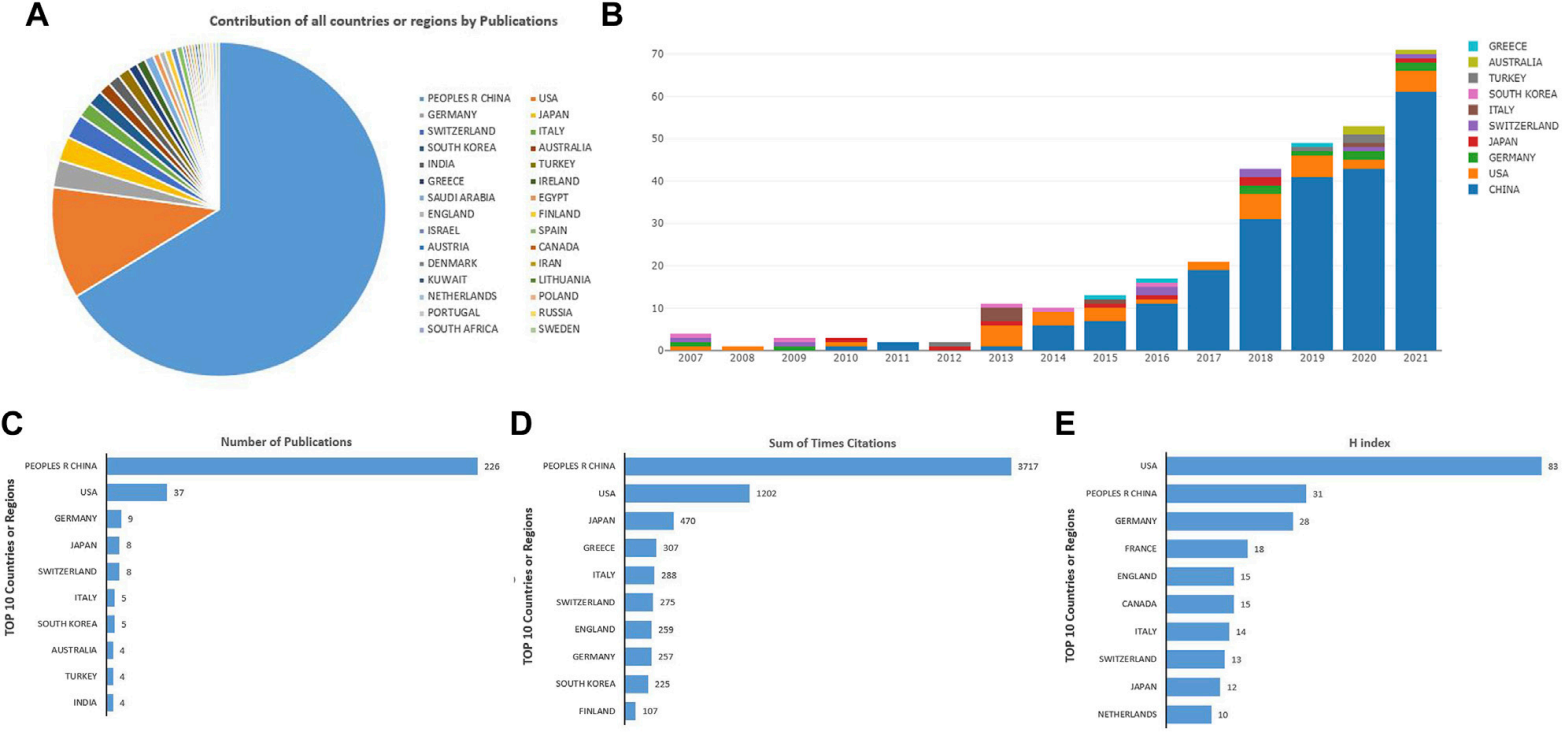
**(A)** Contribution of all countries by Publications. **(B)** The distribution of the bibliographic records per year of the top ten countries on oxidative stress in intervertebral disc degeneration. **(C–E)** Total number of publications, sum of total citations, and H-index of top ten countries on oxidative stress in intervertebral disc degeneration.

A total of 30 countries were retrieved with publications on oxidative stress in IDD. China and the United States were in a dominant position (Figure 2A) and first published articles regarding this field in 2010 and 2007, respectively. Only the United States contributed publications in 2008, and there were seven countries contributing publications in 2020 (Figure 2B). China had contributed 226 articles (78.20%) at the top. The United States was the second contributing country with 37 articles (12.80%), followed by Germany with nine articles (3.11%), both Japan and Switzerland with eight articles (2.77%) (Figure 2C). China ranked first in the sum of times citations (3,717), followed by the United States (1,202), Japan (470), Greece (307) and Italy (288) (Figure 2D). Meanwhile, the H-index of the United States was 83 in the first place. China was the second with 31, followed by Germany (28), France (18) and England (15) (Figure 2E).

### Top 20 most cited articles on oxidative stress in intervertebral disc degeneration

This search collected 289 articles from 2007 to 2021 in WOS. The top 20 most cited articles on oxidative stress in IDD are shown in Table 1, including first author, article title, journal of publication, year of publication, the total number of citations, and average citations. The total citations of the top 20 articles ranged from 64 to 217, and the average citations ranged from 4.06 to 24.29. The top article had 217 citations and was published in 2013 by Vo NV (Vo et al., 2013), followed by Vo NV (Vo et al., 2016) with 170 citations in 2016 and Wang F (Wang et al., 2016) with 169 citations in 2016. The oldest article was published by Nerlich AG (Nerlich et al., 2007) in 2007, and the most recent article in the top 20 was published in 2019 by Ren, J (Ren et al., 2019).

**TABLE 1:** Top 20 most cited articles on oxidative stress in intervertebral disc degeneration from 2007 to 2021.

First author	Article title	Journal	Publication year	Total citations	Average citations
Vo, NV	Expression and regulation of metalloproteinases and their inhibitors in intervertebral disc aging and degeneration	Spine journal	2013	217	21.70
Vo, NV	Molecular mechanisms of biological aging in intervertebral discs	Journal of orthopaedic Research	2016	170	24.29
Wang, F	Aging and age-related stresses: a senescence mechanism of intervertebral disc degeneration	Osteoarthritis and cartilage	2016	169	24.14
Chen, DH	Metformin protects against apoptosis and senescence in nucleus pulposus cells and ameliorates disc degeneration *in vivo*	Cell death and disease	2016	135	19.29
Kadow, T	Molecular basis of intervertebral disc degeneration and herniations: what are the important translational questions?	Clinical orthopaedics and related research	2015	130	16.25
Dimozi, A	Oxidative stress inhibits the proliferation, induces premature senescence and promotes a catabolic phenotype in human nucleus pulposus intervertebral disc cells	European cells and materials	2015	127	15.88
Feng, CC	Disc cell senescence in intervertebral disc degeneration: causes and molecular pathways	Cell cycle	2016	121	17.29
Nasto, LA	Mitochondrial-derived reactive oxygen species (ROS) play a causal role in aging-related intervertebral disc degeneration	Journal of orthopaedic research	2013	115	11.50
Chen, JW	The responses of autophagy and apoptosis to oxidative stress in nucleus pulposus cells: implications for disc degeneration	Cellular physiology and biochemistry	2014	114	12.67
Suzuki, S	Excessive reactive oxygen species are therapeutic targets for intervertebral disc degeneration	Arthritis research and therapy	2015	107	13.38
Kim, KW	Senescence mechanisms of nucleus pulposus chondrocytes in human intervertebral discs	Spine journal	2009	105	7.50
Ryhanen, T	Crosstalk between Hsp70 molecular chaperone, lysosomes and proteasomes in autophagy-mediated proteolysis in human retinal pigment epithelial cells	Journal of cellular and molecular Medicine	2009	103	7.36
Musumeci, G	Age-related degeneration of articular cartilage in the pathogenesis of osteoarthritis: molecular markers of senescent chondrocytes	Histology and histopathology	2015	89	11.13
Cheng, YH	Thermosensitive chitosan-gelatin-glycerol phosphate hydrogel as a controlled release system of ferulic acid for nucleus pulposus regeneration	Biomaterials	2011	84	7.00
Krupkova, O	Stability of (-)-epigallocatechin gallate and its activity in liquid formulations and delivery systems	Journal of nutritional biochemistry	2016	78	11.14
Ren, J	Recent progress regarding kaempferol for the treatment of various diseases	Experimental and therapeutic medicine	2019	70	17.50
Ouyang, ZH	The PI3K/Akt pathway: a critical player in intervertebral disc degeneration	Oncotarget	2017	70	11.67
Yang, W	Interleukin-1 beta in intervertebral disk degeneration	Clinica chimica acta	2015	68	8.50
Nerlich, AG	Immunomorphological analysis of RAGE receptor expression and NF-kappa B activation in tissue samples from normal and degenerated intervertebral discs of various ages	Signal transduction pathways, Pt D: inflammatory signaling pathways and neuropathology	2007	65	4.06
Jiang, W	SIRT1 protects against apoptosis by promoting autophagy in degenerative human disc nucleus pulposus cells	Scientific reports	2014	64	7.11

### Contribution of authors, organizations, and countries

1456 authors, 352 organizations, and 30 countries contributed to this field. Table 2 showed the top author with the most publications was Wang K (*n* = 17) from Tongji Medical College (Yin et al., 2021), the second was Shao ZW (*n* = 16) from Tongji Medical College (Chen S. et al., 2020), followed by Wang XY (*n* = 15) from The Second Affiliated Hospital of Wenzhou Medical University (Hu S. et al., 2021), Yang C (*n* = 12) from Tongji Medical College (Song et al., 2021), Zhang Y (*n* = 12) from Third Military Medical University (Xu et al., 2021). Of the 352 organizations, Huazhong University of Science Technology, Shanghai Jiao Tong University, Wenzhou Medical University, Zhejiang University, and Sun Yat Sen University had contributed 31, 21, 20, 15, and 12 publications, respectively. The top five countries with the most publications were China (*n* = 226), the United States (*n* = 37), Germany (*n* = 9), Japan (*n* = 8), and Switzerland (*n* = 8). The corresponding records, H-index, total citations, and average citations of the top five authors, organizations, and countries were shown in Table 2.

**TABLE 2:** The top five high-yield authors, organizations, and countries on oxidative stress in intervertebral disc degeneration from 2007 to 2021.

Category	Rank	Items	Records	H-index	Total citations	Average citations
Author	1	Wang K, Tongji Medical College	17	10	324	19.06
	2	Shao ZW, Tongji Medical College	16	10	260	16.25
	3	Wang XY, The Second Affiliated Hospital of Wenzhou Medical University	15	11	406	27.07
	4	Yang C, Tongji Medical College	12	7	219	18.25
	4	Zhang Y, Third Military Medical University	12	7	287	23.92
Organization	1	Huazhong University of Science Technology	31	13	498	16.06
	2	Shanghai Jiao Tong University	21	11	390	18.57
	3	Wenzhou Medical University	20	13	469	23.45
	4	Zhejiang University	15	7	239	15.93
	5	Sun Yat Sen University	12	9	193	16.08
Country	1	CHINA	226	32	3,717	16.45
	2	United States	37	18	1,202	32.49
	3	Germany	9	7	257	28.56
	4	Japan	8	7	470	58.75
	4	Switzerland	8	7	275	34.38

### Contribution of research directions, funds, and journals

There were 36 research directions, 315 funds, and 122 journals contributing to the publications on oxidative stress in IDD. The top ten high-yield research directions were presented in Table 3. Cell Biology occupied the most records (*n* = 111), the highest H-index of 26, the highest total citations (*n* = 2091) and average citations (*n* = 18.84) (Cheng et al., 2021). Rheumatology occupied the most average citations (*n* = 43.40). Orthopedics had the most highly cited papers (*n* = 2), followed by Research Experimental Medicine, Pharmacology Pharmacy, and Rheumatology. In addition, National Natural Science Foundation of China had the most records (*n* = 143), the highest H-index of 30, the highest total citations (*n* = 2,852) and average citations (*n* = 19.94) (Shi et al., 2021). National Institutes of Health was the second with records (*n* = 27), H-index of 16, the total citations (*n* = 1,044) and average citations (*n* = 38.67). National Institute of Arthritis Musculoskeletal Skin Diseases occupied the most average citations (*n* = 50.19). Four funds had highly cited papers, including National Natural Science Foundation of China, National Institutes of Health, United States Department of Health Human Services, and National Institute of Arthritis Musculoskeletal Skin Diseases (Table 4). Furthermore, Oxidative Medicine and Cellular Longevity occupied the most records (*n* = 29), the highest H-index of 9, the highest total citations (*n* = 273) and average citations (*n* = 9.41) (Hu Y. et al., 2021; Sun et al., 2021). Journal of Orthopaedic Research had the most average citations (*n* = 59.11). Both Journal of Orthopaedic Research and Biomedicine Pharmacotherapy had one highly cited paper. Oxidative Medicine and Cellular Longevity had the highest impact factor of 6.543 in 2022. The impact factors of all the ten journals were greater than 3.314 (Table 5).

**TABLE 3:** Top 10 research directions with the most publications on oxidative stress in intervertebral disc degeneration from 2007 to 2021.

Research directions	Records	%	Total citations	Average citations	Highly cited papers	H-index
Cell biology	111	38.41	2091	18.84	0	26
Research experimental medicine	66	22.84	917	13.89	1	19
Orthopedics	49	16.96	1855	37.86	2	23
Biochemistry molecular biology	43	14.88	700	16.28	0	14
Pharmacology pharmacy	27	9.34	344	12.74	1	12
Neurosciences neurology	24	8.30	751	31.29	0	13
Oncology	11	3.81	171	15.55	0	7
Geriatrics gerontology	10	3.46	188	18.80	0	7
Physiology	10	3.46	218	21.80	0	7
Rheumatology	10	3.46	434	43.40	1	8

**TABLE 4:** Top 10 funds with the most publications on oxidative stress in intervertebral disc degeneration from 2007 to 2021.

Funds	Records	%	Total citations	Average citations	Highly cited papers	H-index
National natural science foundation of china (NSFC)	143	49.48	2,852	19.94	1	30
National institutes of health (NIH-USA)	27	9.34	1,044	38.67	1	16
United states department of health human services	27	9.34	1,044	38.67	1	16
Natural science foundation of zhejiang province	21	7.27	525	25.00	0	11
National institute of arthritis musculoskeletal skin diseases (NIAMS)	16	5.54	803	50.19	1	11
National key research and development program of china	12	4.15	190	15.83	0	6
Fundamental research funds for the central universities	10	3.46	82	8.20	0	5
China postdoctoral science foundation	8	2.77	53	6.63	0	4
National natural science foundation of guangdong province	8	2.77	129	16.13	0	6
National key R D program of china	7	2.42	74	10.57	0	3

**TABLE 5:** Top 10 journals with the most publications on oxidative stress in intervertebral disc degeneration from 2007 to 2021.

Journals	Records	%	Total citations	Average citations	Highly cited papers	H-index	Impact factor (2022)
Oxidative medicine and cellular longevity	29	10.04	273	9.41	0	9	6.543
Bioscience reports	9	3.11	86	9.56	0	5	3.840
Journal of cellular and molecular medicine	9	3.11	256	28.44	0	7	5.310
Journal of orthopaedic research	9	3.11	532	59.11	1	9	3.494
Life sciences	9	3.11	165	18.33	0	8	5.037
Biomedicine pharmacotherapy	8	2.77	136	17.00	1	7	6.529
Spine	7	2.42	174	24.86	0	5	3.468
Biomed research international	6	2.08	7	1.17	0	1	3.411
European review for medical and pharmacological sciences	6	2.08	30	5.00	0	4	3.507
European spine journal	6	2.08	80	13.33	0	4	3.314

### Co-authorship analysis of publications regarding on oxidative stress in intervertebral disc degeneration

**FIGURE 3:**
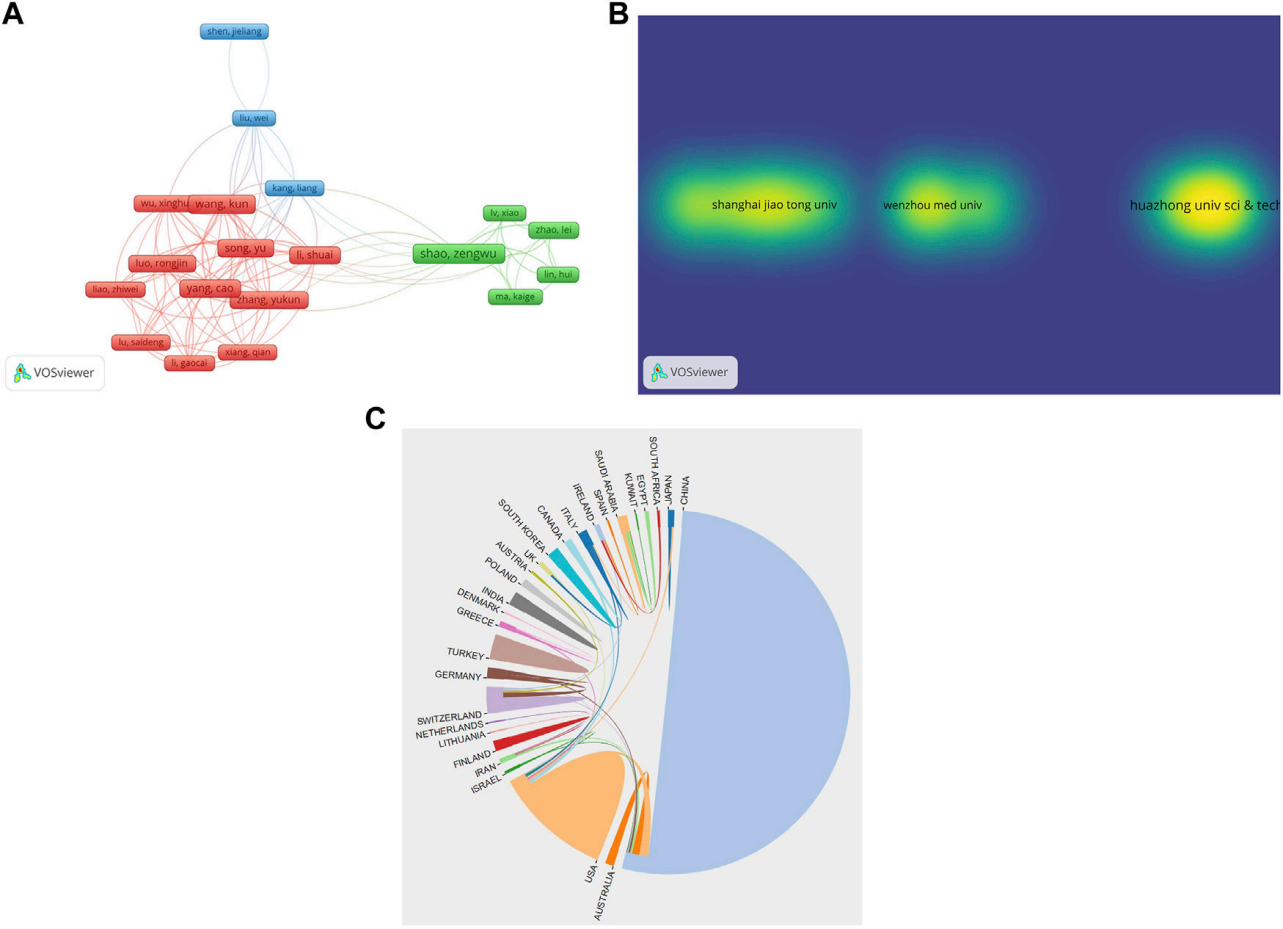
The co-authorship analysis of **(A)** authors, **(B)** organizations, and **(C)** countries on oxidative stress in intervertebral disc degeneration. (The size of the frames represents the proportion of the author in the analysis. The larger the frames, the greater the contribution. The line between the frames represents the connection between the authors. The more or thicker the line, the stronger the connection. The color of the area where organization is located represents the connection between organizations. The darker the color, the closer the collaboration organization; the larger the area, the greater the contribution.)

Both Song Y and Yang C had the most co-authorship strength (total link strength = 81) with 11 documents and 200 citations (Lu et al., 2021; Wang B. et al., 2021), followed by Wang K (total link strength = 77) with 12 documents and 217 citations (Chen Y. et al., 2020), Li S (total link strength = 72) with 10 documents and 235 citations (Hua et al., 2020) (Figure 3A). Moreover, the closest collaboration organization was Wenzhou Medical University (total link strength = 23) with 20 documents and 469 citations. The second organization was Zhejiang Provincial Key Laboratory of Orthopaedics (total link strength = 15) with seven documents and 288 citations, and the third was Chinese Orthopaedic Regenerative Medicine Society (total link strength = 14) with six documents and 239 citations (Figure 3B). Besides, the strongest collaborative country was the United States (total link strength = 18) with 37 documents and 1,202 citations, followed by China (total link strength = 13) with 226 documents and 3,717 citations, and Germany (total link strength = 10) with nine documents and 257 citations (Figure 3C).

### Co-citation analysis of publications regarding on oxidative stress in intervertebral disc degeneration

**FIGURE 4:**
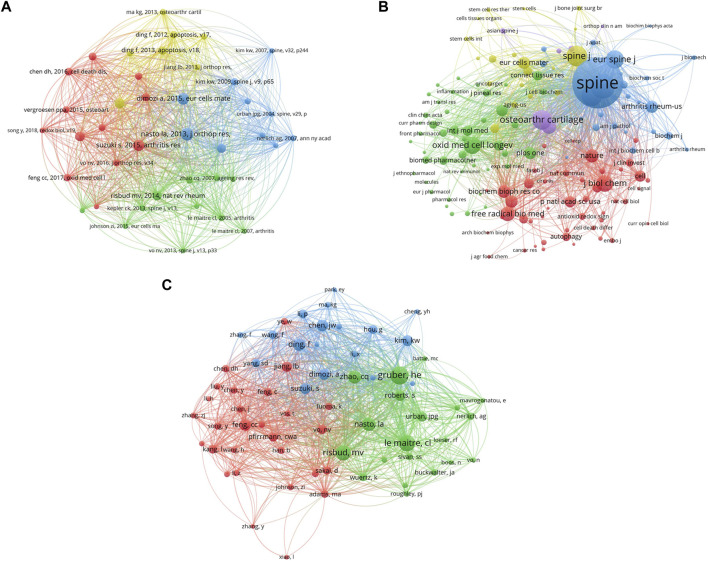
The co-citation analysis of **(A)** references, **(B)** journals, and **(C)** authors on oxidative stress in intervertebral disc degeneration. (A point in the figure represents one reference, journal, and author, respectively. The color of the point represents different clusters, and the size of the point represents the number of citations for each reference, journal, and author, respectively. The more the number, the larger the point. The connection between the two points represents two papers are jointly cited by another paper, and the length of the connection between the two points represents the correlation between two articles. The shorter the line, the stronger the correlation.)

The most co-citation reference (*n* = 73) was “Mitochondrial-derived reactive oxygen species (ROS) play a causal role in aging-related intervertebral disc degeneration,” published by Nasto LA on Journal of Orthopaedic Research in 2013 (Nasto et al., 2013). The second reference (*n* = 65) was “Oxidative stress inhibits the proliferation, induces premature senescence and promotes a catabolic phenotype in human nucleus pulposus intervertebral disc cells,” published by Dimozi A on European Cells and Material*s* in 2015 (Dimozi et al., 2015), and the third reference (*n* = 63) was “Excessive reactive oxygen species are therapeutic targets for intervertebral disc degeneration,” published by Suzuki S on Arthritis Research and Therapy in 2015 (Suzuki et al., 2015) (Figure 4A). On the other hand, the most co-citation journal was Spine (*n* = 1,187) (Chen et al., 2019), followed by Journal of Orthopaedic Research (*n* = 1,187) (Alvarez-Garcia et al., 2017) and Arthritis Research and Therapy (Li et al., 2017) (Figure 4B). Furthermore, the most co-citation author was Gruber, HE (*n* = 138) (Gruber et al., 2013). The second was Le Maitre CL (*n* = 119), and the third was Risbud MV (*n* = 108) (Silagi et al., 2018) (Figure 4C).

### Co-citation timeline of references and burst analysis

**FIGURE 5:**
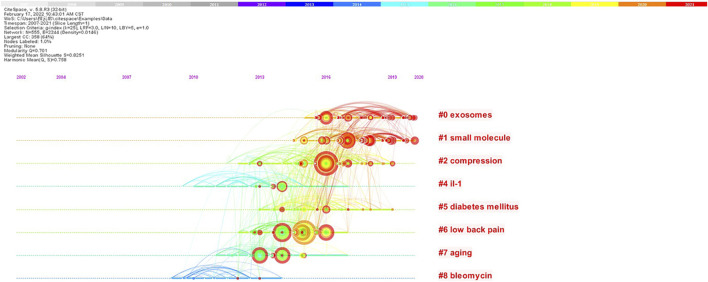
Co-citation timeline of references by keywords on oxidative stress in intervertebral disc degeneration. (The nodes represent the references. The larger the node, the more citations the reference. The colors of the nodes from the inside to the outside correspond to color scale, which represents the total co-citations for the reference in the specific year. The line between two nodes represents two references co-citations. The thicker the line, the more the co-citations. The color of the connection line corresponds to the color mark above, which can reflect the time when two references were first co-cited.)

**FIGURE 6:**
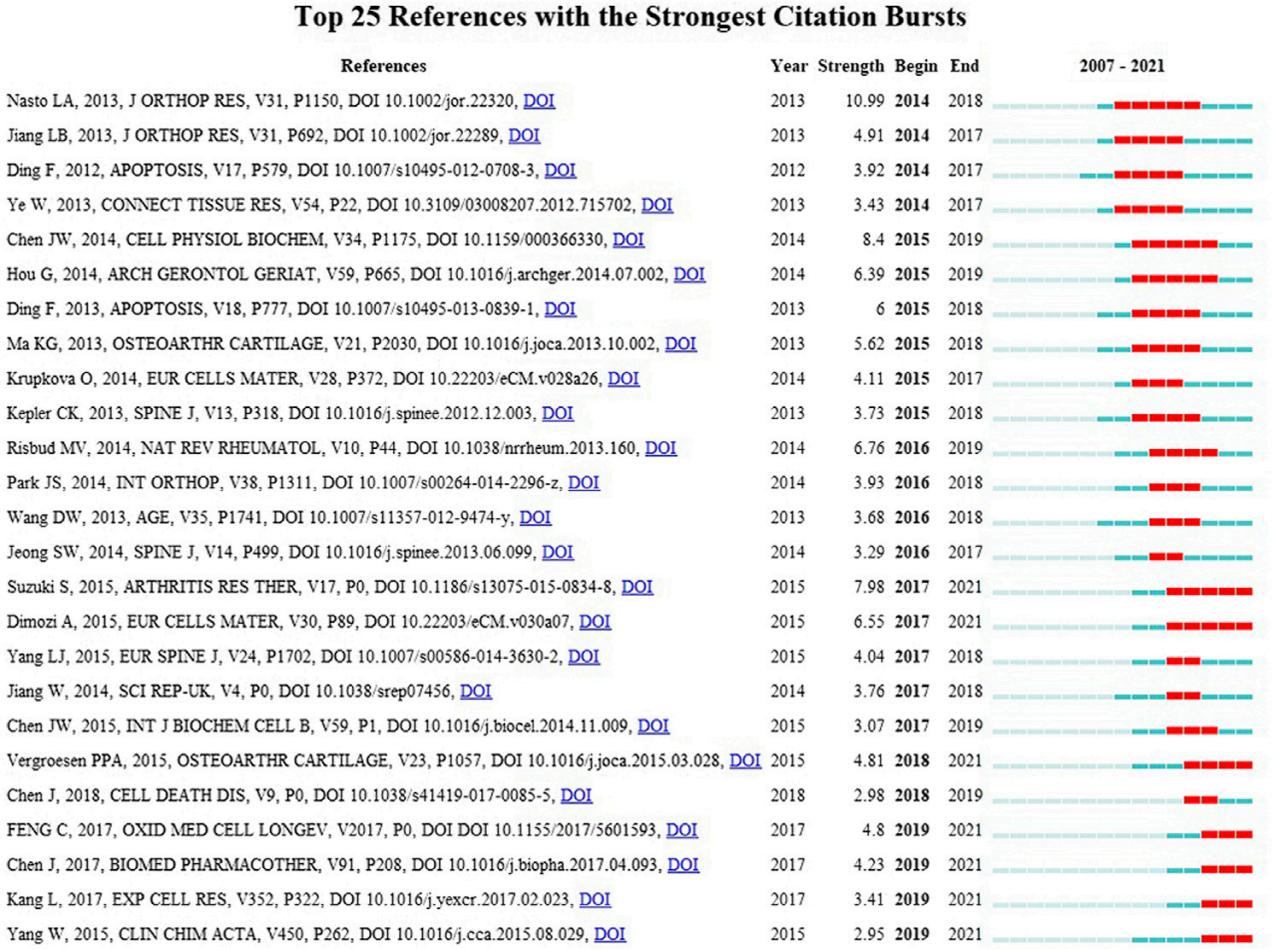
Top 25 references with the strongest citation bursts on oxidative stress in intervertebral disc degeneration.

Co-citation of references for a timeline diagram was drawn by Citespace software (Figure 5). References to the same cluster were arranged on the timeline in chronological order of publication. “Exosomes,” “small molecule,” “compression,” and “low back pain” were the clusters with the most published references. According to the year of publication, “il-1” and “bleomycin” were the clusters with the earliest references. “Exosomes” and “small molecule” were the clusters with the latest references. The top 25 references with the highest burst value were shown in Figure 6 (Ding et al., 2012; Jiang et al., 2013; Ye et al., 2012). Citation burst refers to references with a substantial increase of citations during a short period, reflecting the research focus within a period (Wang Y. et al., 2021).

### Keywords visualization analysis

**FIGURE 7:**
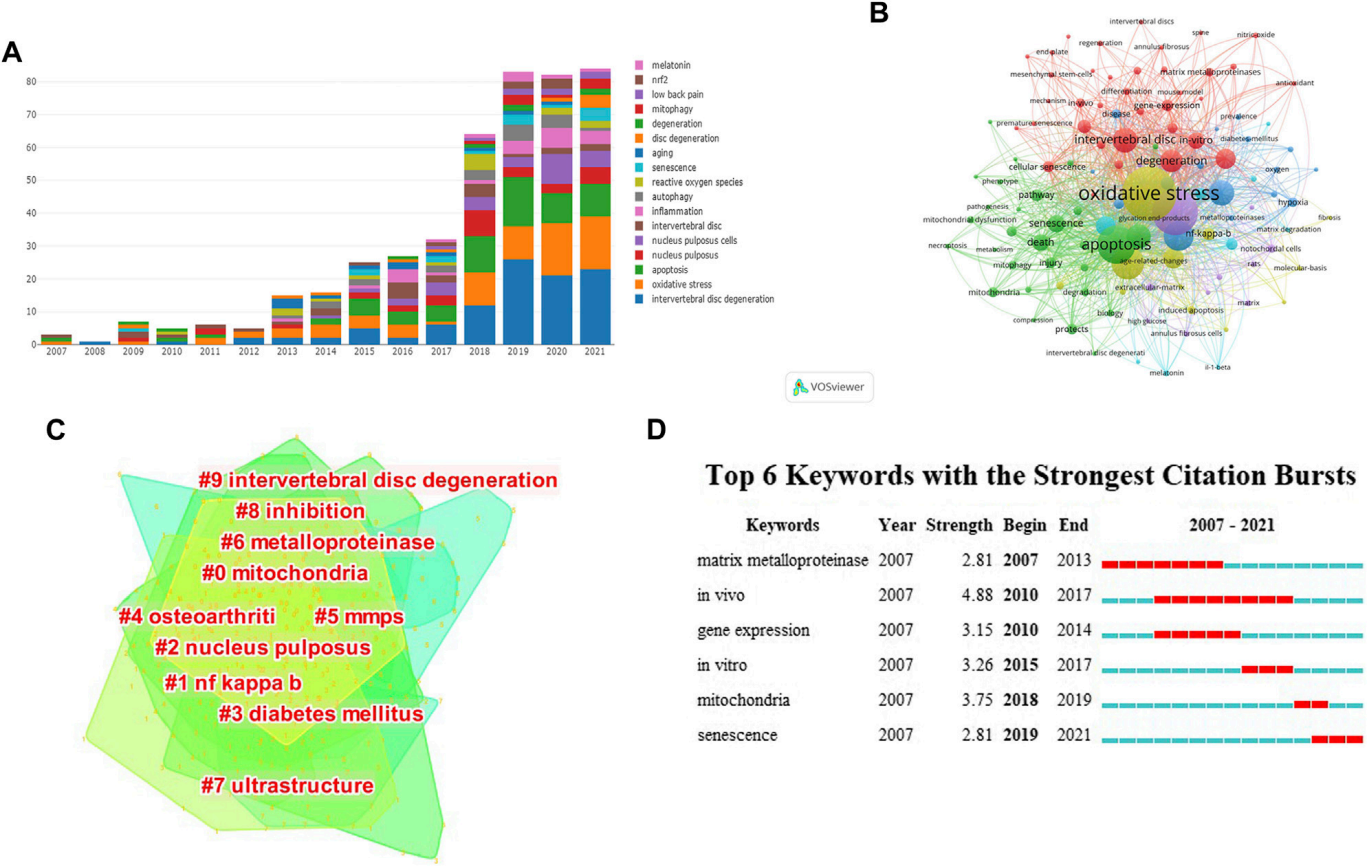
**(A)** The distribution of the bibliographic keywords per year of the top ten countries on oxidative stress in intervertebral disc degeneration. **(B)** Keywords co-occurrence visualization on oxidative stress in intervertebral disc degeneration. **(C)** Key words clusters on oxidative stress in intervertebral disc degeneration. **(D)** Top six keywords with the strongest citation bursts on oxidative stress in intervertebral disc degeneration. (A point in the figure represents a keyword. The color of the point represents different clusters, and the size of the point represents the co-occurrence for each keyword. The more the co-occurrence, the larger the point; # represents different cluster labels, #0 mitochondria, #1 NF-kappa b, #2 nucleus pulposus; #3 diabetes mellitus, #4 Osteoarthriti, #5 MMPS; #6 metalloproteinase, #7 ultrastructure, #8 inhibition; #9 intervertebral disc degeneration.)

Changes in the number of keywords from2007 to 2021 were shown in Figure 7A. There was only one keyword (intervertebral disc degeneration) in 2008, but the number of keywords showed an obvious increase after 2013 and peaked in 2021. Visualization of co-occurrence by keywords was drawn in Figure 7B. The highest occurrence keywords were “oxidative stress” with 183 occurrences (total link strength = 1,169), “intervertebral disc degeneration” with 143 occurrences (total link strength = 939), “apoptosis” with 109 occurrences (total link strength = 809), followed by “nucleus pulposus cells” with 87 occurrences (total link strength = 608), “expression” with 78 occurrences (total link strength = 555).

At the same time, the Log-likelihood rate (LLR) algorithm was used to cluster all keywords and draw a cluster map, a total of 10 Clustering (Figure 7C and Table 6). The smaller the cluster number, the more keywords the cluster contains. The cluster labels were: #0 mitochondria, #1 nf kappa b, #2 nucleus pulposus, #3 diabetes mellitus, #4 Osteoarthriti, #5 mmps, #6 metalloproteinase, #7 ultrastructure, #8 inhibition, #9 intervertebral disc degeneration. Each label is interconnected and developed, not independently existing. The color corresponding to the cluster area indicates the first time that a co-citation appears.

**TABLE 6:** Details of top 10 clusters for researches on oxidative stress in intervertebral disc degeneration from 2007 to 2021.

Cluster no.	Size (n)	Silhouette	Mean (Year)	LSI	LLR	MI
0	65	0.545	2018	intervertebral disc degeneration; glucagon like peptide 1; transcription factor; competitive endogenous RNA; adaptation | oxidative stress; nucleus pulposus cells; mitochondrial dysfunction; activation; honokiol	mitochondria; intervertebral disc degeneration; differentiation; hypoxia; promote	tp53-induced glycolysis and apoptosis regulator; atf4; coculture; AMPK/pgc-1 alpha pathway; cyclosporine a
1	53	0.606	2016	intervertebral disc degeneration; low back pain; nucleus pulposus; targeted therapy; signaling pathway | intervertebral disc; oxidative stress; cell apoptosis; NF-kappa b activation; anti-senescence therapy	NF-kappa b; degeneration; annulus fibrosus cell; intervertebral disk; IDD	Glutathion peroxidase-1; cardiomyocyte hypertrophy; misfolded protein; involvement; cordycepin
2	49	0.626	2014	nucleus pulposus; intervertebral disc; matrix metalloproteinases; replicative senescence; oxidative stress | intervertebral disc degeneration; animal model; magnetic resonance imaging; rhesus monkey; mitochondrial dysfunction	nucleus pulposus; 17 beta estradiol protect; magnetic resonance imaging; DNA damage; animal model	phosphorylation; t1 rho; elderly subject; hyperosmotic stress; high glucose
3	33	0.676	2015	intervertebral disc degeneration; nucleus pulposus; intervertebral disc; primary cell culture; gene expression | oxidative stress; nucleus pulposus cells; autophagic flux; primary cell culture; intervertebral disc	diabetes mellitus; reactive oxygen species; mitoq; bax; Kinsenoside	mitoq; bax; kinsenoside; anti-oxidant; mangiferin
4	33	0.862	2013	intervertebral disc degeneration; noncoding RNA; aerobic metabolism; tissue; degradation | oxidative stress; intervertebral disc; HEME oxygenase-1; noncoding RNA; aerobic metabolism	Osteoarthriti; nlrp3 inflammasome; degradation; gene; hbp1	hbp1; CDDO-EA; oligomeric matrix protein; aerobic metabolism; oxidative phosphorylation
5	31	0.831	2014	intervertebral disc; mitochondrial redox homeostasis; advanced glycation end products; synovial joints; pathomechanisms | intervertebral disc degeneration; advanced glycation end-products; pathomechanisms; carboxymethyl-lysine; metalloproteinases	MMPS; proteomics; aging; controls; advanced glycation end products	controls; advanced glycation end products; pentosidine; sirtuin 3; hemopexin
6	25	0.759	2014	low back pain; metalloproteinase; gene expression; induced inflammation; model | oxidative stress; TNF-alpha; prevalence; inflammatory cytokine; inhibitor	metalloproteinase; senescence markers; induced inflammation; human retina; antioxidant system	senescence markers; induced inflammation; human retina; antioxidant system; inflammatory cytokine
7	23	0.928	2012	oxidative stress; intervertebral disc; enzyme encapsulation; polymer capsules; inflammatory markers | intervertebral disc degeneration; nutrient deficiency; inflammatory markers; intervertebral disc; epigallocatechin gallate	ultrastructure; hydrogen peroxide; doxorubicin; macrophage; oxidative stress markers	doxorubicin; macrophage; oxidative stress markers; iron; age-related macular degeneration
8	18	0.728	2014	intervertebral disc degeneration; nucleus pulposus cells; small molecule; intervertebral disc; death | oxidative stress; mitochondrial function; nucleus pulposus-derived mesenchymal stem cells; PPAR gamma coactivator-1; silent information regulator	inhibition; complement system; naringin; b; premature senescence	complement system; naringin; b; premature senescence; small molecule activator
9	17	0.928	2012	intervertebral disc degeneration; oxidative stress; reactive oxygen species; mitochondral damage; high glucose | nucleus pulposus; extrogen receptor beta; clinical studies; biochemical mechanisms; transcription factor	intervertebral disc degeneration; high glucose concentration; migration inhibitory factor; vo-ohpic; smg1	high glucose concentration; migration inhibitory factor; vo-ohpic; smg1; Wnt/beta-catenin signaling pathway

The top 6 keywords with the highest burst value were demonstrated in Figure 7D. The period occupied by red on the right was the duration of the keywords. According to the burst strength and duration of the keywords, the transformation of domain research direction can be roughly divided into three stages. The first stage was from 2007 to 2014, with the keywords “matrix metalloproteinase,” “*in vivo,*” and “gene expression.” The second stage was from 2015 to 2018, with the keywords “*in vivo,*” “*in vitro,*” and “mitochondria.” The third stage was from 2019 to 2021, with the keywords “mitochondria” and “senescence”.

## Discussion

Bibliometrics mainly collects bibliographic databases and bibliometric features, using the literature database and statistical methods to analyze multiple information in the literature through an all-around way, which helps researchers to timely understand the research direction and development trend of related fields (Xing et al., 2018; Zhang et al., 2013). Scholars can also find current research hotspots and hotspot directions in a specific field by using visual analysis software to analyze the literature further (Wu et al., 2021).

### General information and bibliometric analysis

The number of publications on a specific topic can reflect the popularity in this field. The research regarding oxidative stress in IDD was initially published in 2007. The number of articles published fluctuated around four from 2007 to 2012 and increased rapidly from 2013 to 2021. On the other hand, the significance of a specific topic can be judged by the number of citations (Lin et al., 2020). There was a linear growth in the citation times from 2007 to 2021. The promising future trend on oxidative stress in IDD could be found in Figure 1.

As shown in Figure 2, China was dominant in this field because of the number of publications, far more than that in the United States. Meanwhile, the publications of these two countries account for 91%, which may be due to more attention paid to this field from Chinese and American scholars. China had the most publications and citations. However, the United States had the highest H-index.

### Analysis on top 20 most cited articles about oxidative stress in intervertebral disc degeneration

The more frequent citation an article has, the more influence was considered in the specific field (Marino et al., 2009). It is well known that citation analysis is a systematic method to assess the influence of scientific research (Moed, 2009). As shown in Table 1, the total citation of each article on oxidative stress in IDD was more than 64. The most cited article was a review published by Vo NV in 2013 on Spine Journal, focusing on the expression and regulation of metalloproteinases and their inhibitors in intervertebral disc aging and degeneration. It was concluded that upregulation of MMP and ADAMTS expression and enzymatic activity was implicated in disc extracellular matrix (ECM) destruction, leading to the development of IDD. Future IDD therapeutics depends on identifying specific MMPs and ADAMTSs whose dysregulation results in pathological proteolysis of disc ECM (Vo et al., 2013).

The second most cited article was also published by Vo NV in 2016 on Journal of Orthopaedic Research, paying close attention to molecular mechanisms of biological aging in intervertebral discs. The article showed that imperative given aging was a crucial risk factor for IDD-associated chronic pain and disability, the prevalence of which will undoubtedly be amplified with the growing aging population (Vo et al., 2013).

### Analysis on contribution of authors, organizations, and countries

H-index represents the article in the literature that has been cited at least h times by other researchers, which is a method of estimating an author, organization or country by the number of academic output and the index of academic output level (Hebl et al., 2008). The total number of reference citations refers to the number of times the reference has been cited within a period, an essential indicator for evaluating individual national influence in the specific field (Kalcioglu et al., 2018).

All the top five high-yield authors came from China, consistent with the contribution of China to this important field. Three of them were from Tongji Medical College, showing the great influence of this organization. Similarly, all the top five high-yield organizations belonged to Chinese organizations, including Huazhong University of Science Technology, Shanghai Jiao Tong University, Wenzhou Medical University, Zhejiang University, and Sun Yat Sen University. This result may explain why China was ranked first with a total of 226 records, which was far more than that in other countries (Table 2).

### Analysis on contribution of research directions, funds, and journals

The results of WOS can be used to analyze the published literature in many different views, including the contribution of research directions, funds, and journals. Table 3 to Table 5 presented that Cell Biology, Research Experimental Medicine, and Orthopedics were the most hotspots, which will help orthopedic physicians better grasp the appropriate research directions in the future. Additionally, National Natural Science Foundation of China, National Institutes of Health in United States, and United States Department of Health Human Services were the most high-yield funds. One belongs to China, and the other two belongs to the United States, which may be a good reason to explain why China and the United States were dominant in this field.

In addition, identifying the dominant journals in a specific topic can guide scholars to construct scientific achievement. Oxidative Medicine and Cellular Longevity, Bioscience Reports, and Journal of Cellular and Molecular were the most high-yield journals. Especially for Oxidative Medicine and Cellular Longevity, it had 29 publications (10.04%), far more than other journals. Focusing on high-yield journals can help scholars access the most authoritative knowledge framework and the orientation of manuscript submitting. The publishers of the two journals belong to England, while the rest one is from the United States. Researchers can benefit from this important information and realize the deficiencies when high-level articles appear (Liu et al., 2021).

### Co-authoring and Co-citation analysis

VOSviewer software was widely used for cooperation network analysis of authors, organizations, and countries; co-citation analysis of references, journals, and authors (Yu et al., 2020). Song Y, Yang C, and Wang K were the authors with the most total link strength, and each of their strength was more than 70, which indicated that the cooperation between the authors was strong (Figure 3A). Similarly, Wenzhou Medical University, Zhejiang Provincial Key Laboratory of Orthopaedics, and Chinese Orthopaedic Regenerative Medicine Society were organizations with the most total link strength. However, each strength was no more than 25, which may indicate that the cooperation between the organizations was not strong enough (Figure 3B).

As shown in Figure 3C, the strongest collaborative country was the United States, followed by China and Germany. Nevertheless, each strength was no more than 20, which may also indicate that the cooperation between the countries was not strong enough. The citation of the top ten references was above 30, and the most citation was 73. Spine, Journal of Orthopaedic Research, and Arthritis Research and Therapy were the journals with the most citations, and each of the citations was more than 333. Furthermore, Gruber, HE, Maitre CL, and Risbud MV were the authors with the most citations, and each of the citations was over 108. (Figure 4).

### Research hotspots and trends analysis

The keywords cluster analysis was a positive method to evaluate the research hotspots on oxidative stress in IDD. The first cluster was mitochondria. Based on the existing knowledge, mitochondrial structural and functional disruption has been observed in degenerated nucleus pulposus (NP) cells and intervertebral discs. Multi-level and well-orchestrated strategies, mitochondrial quality control, are involved in maintaining mitochondrial integrity, mitochondrial proteostasis, the mitochondrial antioxidant system, mitochondrial dynamics, mitophagy, and mitochondrial biogenesis (Song et al., 2021). Accumulating evidence has demonstrated that disrupted mitochondrial dynamics were also closely related to mitochondrial dysfunction and oxidative stress in the IDD process (Xu et al., 2019). The second cluster was nuclear factor-kappa B (NF-kB), a unique nuclear transcription factor widely present in the cytoplasm of higher eukaryotes. NF-kB is primarily present in the form of P65-P50 dimer and is associated with inhibitor IkB binds in an inactive state (Biswas et al., 2001). A study has shown that the expressions of NF-kB, MIF, IL-6, and TNF-α in intervertebral disc tissue in patients with disc herniation were increased and related to the degree of disc herniation. It may play an important role in the pathophysiological process of disc herniation (Liang et al., 2018). The third cluster was nucleus pulposus. The intervertebral disc is composed of the inner NP and outer annulus fibrosus, which functions as the load-bearing and buffering unit of the spine. In the inner disc, NP cells mainly produce and maintain the ECM. It is accepted that IDD progression is initiated and accelerated by the depletion of NP cells and the degradation of ECM (Vergroesen et al., 2015). One research showed that the delivery of exosomes derived from bone marrow mesenchymal stem cells could modulate endoplasmic reticulum stress and inhibit excessive NP cell apoptosis during IDD (Liao et al., 2019).

The keyword burst analysis could effectively grasp the research trends on oxidative stress in IDD. The latest burst was senescence, which was classically defined as an irreversible cell-cycle arrest caused by telomere uncapping or various external stimuli. NP cell senescence was determined in degenerative discs, and the number of studies discussing NP cell senescence increases every year (Feng et al., 2016). However, to date, the knowledge of characteristics of senescent cells has been expanded (van Deursen, 2014). Cell senescence in IDD results from the general aging process and the structure and tissue specificity of discs (Zhang et al., 2020). A recent study proved that Urolithin A could activate the SIRT1/PGC-1α signaling pathway to protect mitochondrial function and alleviate cell senescence and IDD *in vivo* and vitro (Shi et al., 2021).

## Limitations

Bibliometric analysis has been widely used to measure the impact of articles in recent years. However, there are still some limitations in this method. First, we only used the core collection of WOS for searching literature, which is a single database. The more databases we use, the more information we can get and analyze. Other databases such as InCites and MEDLINE should be considered in future. Second, the main language of WOS is English. Articles written in other languages are excluded, which means some relevant articles to be not included. Third, the citation number of each literature is time-dependent. Different times to search the articles, different citations may obtain. However, the trend of citation number of each literature is nearly the same.

## Conclusion

Research on oxidative stress in IDD was in a rapid development stage. Annual publications on this field have been continually increasing since 2013, especially in the last 2 years. China has contributed most to this field, and the United States has also played a significant role with the highest H-index. The most high-yield author, organization, country, research directions, funds, and journals were Wang K from Tongji Medical College, Huazhong University of Science Technology, China, Cell Biology, National Natural Science Foundation of China, Oxidative Medicine and Cellular Longevity, respectively. “Mitochondria,” “NF-kB,” and “NP” were the largest cluster terms, which indicates the future hotspots on oxidative stress in IDD. “Mitochondria” and “senescence” were the latest two burst terms in recent years, which indicates the research trends on oxidative stress in IDD. Our results could benefit scholars by quickly grasping research hotspots and trends, providing objective insight into further research.

## Data Availability

The raw data supporting the conclusion of this article will be made available by the authors, without undue reservation.
